# Intraosseous Basivertebral Nerve Ablation in the Treatment of Vertebrogenic Back Pain Secondary to Advanced Osteopenia: A Case Report

**DOI:** 10.7759/cureus.81457

**Published:** 2025-03-30

**Authors:** Daniel J Lopez, Rafael A Peralta, Leon Anijar

**Affiliations:** 1 Pain Medicine, University of Florida, Gainesville, USA; 2 Emergency Medicine, HCA Westside Regional Medical Center, Plantation, USA; 3 Pain Medicine, Spine and Wellness Centers of America, Aventura, USA

**Keywords:** back pain, basivertebral nerve ablation, osteopenia, osteoporosis, pain management

## Abstract

Low back pain in advanced osteopenia and osteoporosis continues to challenge the pain provider in offering effective analgesia for this growing population. Basivertebral nerve ablation is proven to benefit vertebrogenic back pain. A relative contraindication to basivertebral nerve ablation is metabolic bone disease.

An 81-year-old male with a medical history of advanced osteopenia, with a T-score of -2.2, presented to an outpatient pain clinic with chronic low back pain. MRI of the spine demonstrated Type 1 and Type 2 Modic changes of the L3, L4, and L5 vertebral endplates, without evidence of fracture. The patient underwent an intraosseous basivertebral nerve ablation of the L3, L4, and L5 vertebrae, and follow-up appointments up to six months demonstrated a reduction in the patient's Numeric Pain Rating Scale by 6 and an improvement in quality of life.

We successfully performed intraosseous basivertebral nerve ablations in a patient with advanced osteopenia, for the treatment of vertebrogenic back pain.

## Introduction

Low back pain is one of the most common causes of disability, with a lifetime prevalence as high as 70% in the global population [1,2]. With the rise in overall life expectancy, metabolic bone disease leading to degeneration in the form of osteopenia and osteoporosis has proportionally increased.

Among patients with osteopenia, a hallmark of disease progression is chronic low back pain. Pain management and disease-altering therapy remain a difficult task in this patient population, leading to costly outcomes secondary to extensive medical therapy, interventional therapy, and often, spinal surgery that may require repeat operations [3-5].

Among the many causes of chronic low back pain, a few etiologies that exist are muscular-related pain, facet joint pain, discogenic pain, spinal canal stenosis, failed back surgery syndrome, and sacroiliitis [6]. A common, yet misdiagnosed, pathologic cause of low back pain can be localized to vertebral endplate damage secondary to progressive disc degeneration, known as vertebrogenic back pain.

The basivertebral nerve, a branch of the sinuvertebral nerve, transmits these pain signals from the affected vertebral body endplates [7]. A novel, minimally invasive procedure in which the basivertebral nerve is targeted and ablated using radiofrequency has been proven highly effective for pain control in patients suffering from vertebrogenic back pain.

Although this treatment option is effective, the study trials utilizing the aforementioned intervention excluded patients with metabolic bone disease from this procedure due to its relative contraindication secondary to bone fragility [8]. We present a case that demonstrates the successful use of an intraosseous basivertebral nerve radiofrequency ablation of the third, fourth, and sixth lumbar vertebrae (L3, L4, and L5) in a patient suffering from vertebrogenic back pain secondary to advanced osteopenia.

## Case presentation

**Figure 1:**
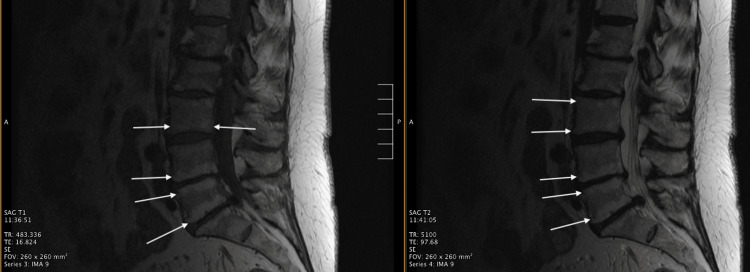
MRI sagittal T1 (left) and T2 (right)-weighted images demonstrating (white arrows) a mix of both Type 1 and Type 2 Modic endplate changes on the L2, L3, L4, L5, and S1 vertebral bodies. Note that we only performed the procedure at L3, L4, and L5.

An 81-year-old male with a past medical history of progressive, advanced osteopenia presented to our outpatient pain clinic with worsening chronic low back pain. The patient denied current or former tobacco use and followed a treatment regimen with his rheumatologist, consisting of lifestyle modifications, calcium, vitamin D, and oral bisphosphonates. The patient's T-score measured -2.2 on bone mineral density (BMD) by dual-energy X-ray absorptiometry (DXA). Medical history was negative for vertebral fragility compression fractures. Physical exam was significant for axial low back pain aggravated by forward flexion while standing. The patient had negative tenderness on palpation of the lumbar spinous processes, interspinous spaces, and paraspinal regions. Despite completing over a year of medical therapy with anti-inflammatories, gabapentinoids, bisphosphonates, and physical therapy, the patient continued to have medically refractory low back pain. The patient’s Numeric Pain Rating Scale (NPRS) for low back pain was 9/10. The patient's Oswestry Disability Index (ODI) score was 29, indicating moderate disability due to the patient's low back pain. An MRI of the spine was performed and was significant for bony degeneration of the spine, consistent with Type 1 and Type 2 Modic changes of the third lumbar vertebral body inferior endplate, fourth lumbar vertebral body superior and inferior endplates, and the fifth lumbar vertebral body superior endplate (Figure 1). After a thorough discussion regarding treatment options for the patient’s vertebrogenic back pain, a basivertebral nerve ablation of the third, fourth, and fifth lumbar vertebral bodies was offered.

The basivertebral nerve ablation occurred at an ambulatory surgery center using monitored anesthesia care. Aseptic precautions, in full surgical attire with sterile technique, were conducted. The patient was placed in the prone position during the entire procedure. Using a radiolucent table, a true anteroposterior (AP) view of the thoracolumbar spine was established, with the C-arm X-ray machine positioned directly over the patient. An intraosseous basivertebral nerve ablation of the third lumbar (L3) vertebra was performed as follows: The right L3 pedicle was identified under fluoroscopy. A transpedicular approach was performed to access the vertebral body as follows: a small incision was made on the skin at the 2 o’clock position, approximately 2 cm lateral to the right pedicle. The manufacturer’s introducer cannula with straight stylet was inserted at the incision site at a 30-degree angle from the axial plane, and the pedicle was accessed under fluoroscopic guidance by advancing the cannula in a lateral-to-medial trajectory until the pedicle was contacted. The cannula with straight stylet was advanced through the pedicle to create a channel while using caution to stay within the cortical bone and was stationed just prior to the posterior portion of the vertebral body. The position was verified orthogonally with AP and lateral fluoroscopic views. 

**Figure 2:**
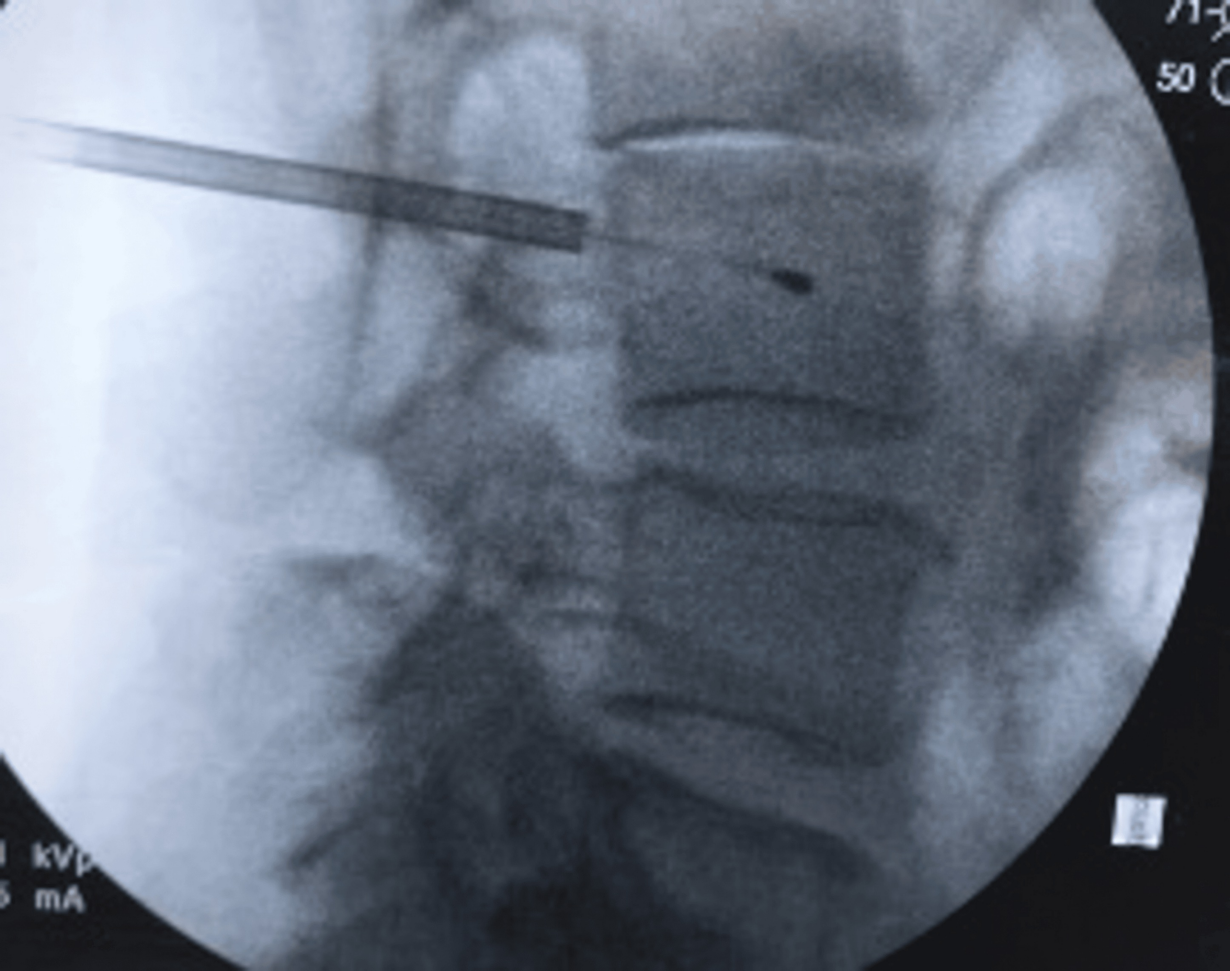
Lateral fluoroscopic image demonstrating the radiofrequency ablation probe in the L3 vertebral body during the basivertebral nerve ablation process.

The straight stylet was removed, and the manufacturer's curved stylet was inserted into the cannula and advanced to reach the trunk of the basivertebral nerve, roughly 40% from posterior to anterior and 50% from inferior to superior in the vertebral body. The curved stylet was removed, and the manufacturer’s bipolar radiofrequency probe was then inserted through the curved cannula and stationed in the center of the vertebral body, roughly 1.5 cm anterior to the posterior vertebral body wall, to avoid the risk of thermal ablation of the spinal canal nerve structures. The position was confirmed with AP and lateral fluoroscopic views. The curved cannula sheath was then slowly retracted, and a lateral fluoroscopic view showed the radiofrequency probe at the final destination, positioned in the L3 vertebral body (Figure 2). Bipolar radiofrequency thermal ablation at 85°C for 15 minutes was performed to create an approximate 1 cm spherical ablation area at the basivertebral nerve trunk. The above procedural technique was then performed for the L4 and L5 vertebral bodies. 

At each follow-up appointment post-intervention, the patient reported a significant reduction in his low back pain by 60% at two weeks, six weeks, three months, and six months. Specifically, the patient’s recalculated ODI score at six months was 13, with an overall percent reduction of 55% and a score decrease of 16 points. Additionally, the patient’s NPRS at six months was 3/10, with an overall percent reduction of 60% and a score decrease of 6. The patient endorsed an increased ability to ambulate for longer distances, sleep for longer periods of time, have an enhanced social life, and sit and drive for longer periods of time. This patient continued to follow his treatment regimen for osteopenia per the rheumatologist.

## Discussion

Osteopenia is defined as a progressive decrease in BMD secondary to abnormal osteoclastic and osteoblastic activity [9]. Specifically, the diagnosis is confirmed with a T-score range from -1 to -2.5 on DXA bone scans. Old age continues to be the primary risk factor, hence the increasing prevalence of osteopenia due to the growing global aging population. Chronic low back pain from decreased BMD is often secondary to vertebral body fragility fractures, although this finding is more prominent and is the hallmark of osteoporosis or advanced osteopenia. Once a fragility fracture is ruled out, the known etiologies of low back pain remain potential causes for the osteopenic patient. With recent data expanding our knowledge of axial low back pain, vertebrogenic pathology is often found [7]. First described in 1988 by Modic et al., vertebral body endplate pathology can be seen on MRI of the spine, showing Type 1 and Type 2 Modic changes at the vertebral body endplates, secondary to degenerative bone disease [10]. Type 1 and Type 2 Modic changes seen on MRI indicate bone marrow edema, inflammation, and ischemia at the vertebral body endplates and can be a cause of low back pain.

For patients diagnosed with vertebrogenic back pain based on clinical and MRI findings of Type 1 and/or Type 2 Modic changes, an effective interventional procedure for pain management is an intraosseous basivertebral nerve ablation. Of note, Type 3 Modic changes found on MRI do not meet the criteria for the use of this procedure due to endplate bony sclerosis. Based on long-term studies, patients who received an intraosseous basivertebral nerve ablation had a two-year follow-up improvement in their vertebrogenic back pain, with a mean decrease of 23.6 points in ODI scores (8). At five years out from the procedure, the patient's ODI scores remained improved from pre-procedure at a mean decrease of 25.95 [11]. Although this procedure has been proven highly effective for pain control in the setting of vertebrogenic back pain, patients with a diagnosis of osteoporosis with a corresponding T-score of less than -2.5 were excluded from the study.

Our presenting case demonstrates successful basivertebral nerve ablation of the L3, L4, and L5 vertebrae in a patient suffering from advanced osteopenia with a T-score of -2.2. Osteopenia is a progressive disease, and studies have shown that about 10% of patients with advanced osteopenia, as in this case, will progress to osteoporosis in less than one year [12]. Pain control in these patients is difficult due to the progressive pathology of the disease state. Analgesic approaches consist of nonpharmacologic therapy, with the use of vibration training, physical therapy, cognitive-behavioral therapy, and mindfulness training, which offer minimal relief. Non-steroidal anti-inflammatory drugs (NSAIDs) are effective; however, this class of drugs poses a risk in this patient population due to its negative effect on bone metabolism [13]. Other treatment options for the patient with low back pain in the setting of osteopenia or osteoporosis include bisphosphonates and opioids. Opioids, especially in this elderly population, are not first-line due to the wide range of side effects, such as nausea, constipation, respiratory depression, somnolence, and more. When these treatment options either fail to offer analgesia or pose increased risks for initiation in the osteopenic patient with vertebrogenic back pain, this case demonstrates that a basivertebral nerve ablation procedure offers an effective and safe option for pain control.

Hesitance to perform this minimally invasive procedure in this patient population can stem from blunting pain signals from potential future fragility vertebral fractures. Although this thought is convincing, studies have shown that for the majority of these fractures, roughly two-thirds are asymptomatic and are found on incidental imaging [14]. If this is a true concern, perhaps serial abdominal or selective spine radiographs can be performed at follow-up appointments to measure height loss, if any, as well as radiographs if the patient sustains a minor traumatic injury to ensure a fracture did not occur. With any procedure, the risks and benefits should be considered. Once a patient with osteopenia or osteoporosis has demonstrated continued vertebrogenic back pain despite six months of medical therapy in the absence of fragility fractures, the benefits of pain control with the use of basivertebral nerve ablation should be highly considered.

## Conclusions

We successfully performed intraosseous basivertebral nerve ablation on the L3, L4, and L5 vertebrae in a patient with advanced osteopenia, resulting in improvement of their low back pain. Osteopenia is a progressive, degenerative bone disease that can rapidly progress to osteoporosis, and patients with this diagnosis were excluded from the main studies for basivertebral nerve ablation due to the fragility of the bone architecture. However, the literature suggests that pain control is difficult to accomplish in this patient population, and certain pharmacologic treatment strategies, such as NSAIDs and opioids, can speed up the disease progression and have debilitating side effects, respectively. Additionally, concerns with blunting of pain signals from future vertebral fragility fractures for patients who undergo this procedure should be of minimal concern, given that the majority of patients without nerve ablation who sustain a fracture are asymptomatic. Given our successful case, patients suffering from vertebrogenic back pain due to advanced osteopenia or osteoporosis, without evidence of fragility fractures, should be considered for the use of basivertebral nerve ablation. Patients with osteopenia or osteoporosis should not be disqualified from basivertebral nerve ablation therapy for the treatment of low back pain.
